# Born Too Soon: Priorities to improve the prevention and care of preterm birth

**DOI:** 10.1186/s12978-025-02069-z

**Published:** 2025-06-23

**Authors:** Etienne V. Langlois, Amy Reid, Joy E. Lawn, Mary Kinney, Maria El Bizri, José M. Belizán, Anna Gruending, Bo Jacobsson

**Affiliations:** 1 Partnership for Maternal, Newborn and Child Health (PMNCH), World Health Organization (WHO), Geneva, Switzerland; 2 London School of Hygiene & Tropical Medicine, London, UK; 3 School of Public Health, University of the Western Cape, Bellville, South Africa; 4 Global Surgery, University of Cape Town Faculty of Health Sciences, Observatory, Cape Town, South Africa; 5 School of Medicine, University of Rosario, Rosario, Argentina; 6 Partnership for Maternal, Newborn and Child Health (PMNCH), World Health Organization (WHO), Paris, France; 7 Department of Obstetrics and Gynecology, University of Gothenburg, Gothenburg, Sweden; 8 Department of Obstetrics and Gynecology, Western Health Care Region, Sahlgrenska University Hospital, Gothenburg, Sweden

For over 50 years there has been a commitment to multilateralism and funding for science and health. Recent shifts around the world jeopardise the ability of all countries to address existing and new health crises, and specifically threaten hard-won progress in reproductive, maternal, newborn, and child health, including for preterm babies who are particularly vulnerable.

Preterm birth remains a silent emergency of a global scale. Every 2 seconds, a baby is born too soon [1]. Every 40 seconds, one of those babies dies. An estimated 13.4 million babies were born preterm (before 37 weeks of pregnancy) in 2020 - equivalent to nearly one in ten babies being born preterm worldwide [2].

Preterm birth is the leading cause of child deaths, accounting for more than 1 in 5 of all deaths of children occurring before their 5th birthday [3]. From 2010 to 2020, an estimated 152 million babies were born preterm. Yet, rates of preterm birth have remained stagnant, with some regions even witnessing an increase. For preterm survivors, challenges extend far beyond the neonatal period, as they face a higher risk of lifelong health complications, including developmental delays, disabilities, and chronic conditions [4]. Limited advancements in the prevention of preterm births and gaps in the implementation of the care of preterm newborns have contributed to the global slowdown in progress for reducing newborn and child mortality since the adoption of the Sustainable Development Goals (SDGs) in 2015. Hence, progress remains insufficient to achieve the necessary reductions in newborn and child mortality to meet the SDG targets by 2030. In response, the 77^th^ World Health Assembly Resolution on accelerating progress for maternal, newborn, and child health [4] included a strong call for intensified global action to address the root causes and effects of preterm birth.

To fast-track evidence-based implementation on the prevention of preterm birth and care for preterm babies, this supplement expands content from the 2023 report “Born too soon: decade of action on preterm birth” [5]. We are also building on the first Born Too Soon report in 2012 [6], which presented the first ever national and global preterm birth estimates, and the linked *BMC Reproductive Health* supplement (2013), which translated the chapters into academic articles [7]. We have emulated this approach with a supplement in the same journal. The papers in this supplement expand on the chapters in the 2023 edition of Born Too Soon. Each paper presents evidence synthesis based on literature reviews, country case studies about good practices on policy and implementation and lived experiences and community perspectives.

This 2025 supplement anchors preterm birth as a key issue within the continuum of maternal and newborn care, including the prevention of stillbirth. The supplement highlights the importance of women’s sexual and reproductive health and rights (SRHR). It places additional emphasis on adolescent girls, who have an increased risk of preterm birth but often have far less access to the services and care that they need to support their health and well-being. Importantly, a life-course perspective is foundational and recognises the intergenerational impacts of preterm birth. Hence, we place additional emphasis on the follow-up care and support that is needed for survivors of preterm birth and their families.

The 2023 Born Too Soon report [5] was part of a campaign to elevate awareness around the burden, solutions and priorities for preterm birth. This movement for action was spearheaded by the Partnership for Maternal, Newborn and Child Health (PMNCH), the world’s largest alliance for women’s, children’s and adolescents’ health and wellbeing, and harnessed three shifts: power of data, people’s stories and partnerships.

## Priorities to catalyse change

### Power of data and new evidence

The 2010 estimates on preterm birth [6, 8] showed that preterm birth affected every country, including high-income ones, with the USA in the top ten for numbers of babies born too soon. Evidence-based action could save over a million children who die needlessly each year [3]. Equity gaps are enormous, with only 1 in 10 extremely preterm babies (< 28 weeks) surviving in low-income countries, compared to more than 9 in 10 in high-income countries [9]. These inequities in survival are driven by a lack of access to quality care and determine the likelihood of preterm birth, death, and disability. Disparities, however, do persist in high-income countries, where marginalised groups face significant challenges in accessing timely and effective care. Poverty often limits access to essential services such as prenatal care, proper nutrition, and safe living conditions, which are critical for preventing preterm births and managing complications. Systemic racism further exacerbates these issues, with minority groups frequently encountering bias, lower-quality care, and obstacles in navigating healthcare systems, resulting in poorer outcomes for both mothers and babies [10].

Yet, investment in preterm birth prevention and care can unlock more human capital than at any other time across the life course, impacting futures for millions of families and resulting in significant human and economic returns [11, 12].

### Power of people at the centre

These data and large numbers reflect affected individuals and communities – women, babies and their families and healthcare workers – which PMNCH put at the heart of the campaign. An essential dimension of the Born Too Soon movement was a collection of narratives from affected families and other lived experiences, including healthcare workers [13]. Parent and patient groups along with healthcare professional associations have led the charge for preterm birth; in this supplement, we argue for a broad and multi-constituency mobilisation around this agenda, to improve investments in preterm birth across sectors.

### Power of partnerships

The 2023 Born Too Soon report [5], and this supplement were fuelled by engagement of 70 organisations, including 140 individuals from 46 countries. The campaign reached 215 media products across six continents, for a total estimated media reach of 3.47 billion people. With a diverse authorship explicitly including leaders from low- and middle-income countries (LMICs) and young experts, the papers in this supplement position the prevention and care for preterm babies as a cornerstone of integrated and high-quality maternal and newborn care, including stillbirth prevention. The 2023 Born Too Soon report was co-published by PMNCH, WHO, UNICEF and UNFPA, and guided by a high-level Advisory Group. In addition, this supplement has been published as part of the 2025–2026 joint WHO and PMNCH advocacy campaign, linked to the 2025 World Health Day theme: “Healthy Beginnings, Hopeful Futures”, calling for unified action and urgent prioritisation of maternal and newborn health [14].

## Papers in this supplement

**Fig. 1:**
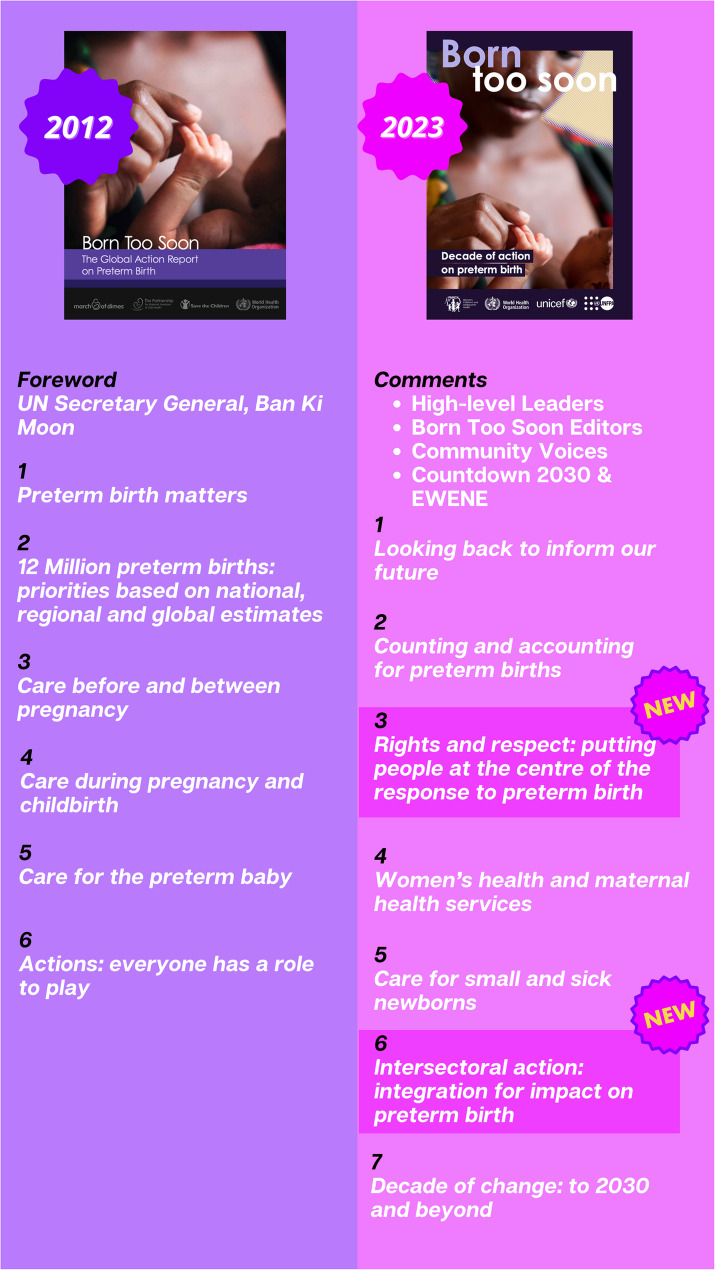
Navigating Born Too Soon 2012 and 2023

This supplement, *Born Too Soon: Progress*,* Priorities*,* and Pivots for Preterm Birth*, expands on the content of the *Born Too Soon 2023* report, adopting a life-course approach that follows women and their newborns over time within a continuum of care (Fig. 1). The supplement consists of seven analysis papers and four commentaries.

We present updated data and evidence from the 2012 report [6], now examined through a broader lens, including new data on preterm birth rates, trends, risk factors, and advances in measurement globally; maternal health and care relevant to preterm prevention, with an emphasis on sexual and reproductive health and rights; care for small and sick newborns; and implementation learning for systems change, extending beyond preterm care alone. Within each paper, content was organised by three domains:

1. progress, particularly in the last decade, related to preterm birth;
2. programmatic priorities based on up-to-date and policy-relevant evidence; and
3. pivots needed to accelerate change in the decade ahead

Five papers in this supplement align to the 2012 set including:

- Paper 1: Learning from the past to accelerate action in the next decade. [15].
- Paper 2: Global epidemiology of preterm birth and drivers for change [16].
- Paper 4: Women’s health and maternal care services, seizing missed opportunities to prevent and manage preterm birth. [17].
- Paper 5: Care for small and sick newborns, evidence for investment and implementation. [18].
- Paper 7: Accelerating change to 2030 and beyond [19].

Two new papers cover novel topics of increasing importance with new evidence that were not included in the 2012 Born Too Soon report (Fig. 1) [6]:

- Paper 3: Progress and priorities for respectful and rights-based preterm birth care. [20].
- Paper 6: Integration of intersectoral interventions for impact on preterm birth. [21].

Overall, these supplement papers emphasise a healthy beginning for vulnerable babies, and the life course approach underlining the follow-up care and support needed for survivors of preterm birth and their families over the course of their lives [15, 18]. The first paper reviews progress during the last decade since the last report, primarily from a policy perspective, and considers the challenges that have hindered advancements and looks ahead, positioning preterm birth as a pivotal issue for driving more rapid and integrated progress for women and children [15]. 

The second paper presents updated national estimates of preterm birth rates for 195 countries, with trends for the last decade emphasising the flatlining of progress for preterm birth rates in every region, with new insights on risk factors. There are positive improvements in measurement and opportunities to more accurately count and account for preterm births and use these data to drive action [16]. 

The third paper focuses on rights and respect for women and their babies, calling for a rights-based approach to respectful care for the mother-baby dyad, especially small and preterm babies who are the most vulnerable. This paper also promotes an enabling environment for healthcare workers with supportive policies and accountability mechanisms [20]. 

The fourth paper focuses on women’s health and maternity services, highlighting the evidence and the importance of seizing missed opportunities in preventing and managing preterm birth within existing care packages. A broader emphasis on sexual and reproductive health and rights is also championed [17]. 

The fifth paper highlights the opportunity for impact and high return on investment with small and sick newborn care, and sets out interventions and innovations for faster implementation with health systems shifts to ensure newborns survive and also thrive and families are at the centre of care. Crucial gaps for infrastructure, devices, data and especially workforce are highlighted [18]. 

The sixth paper focuses on intersectoral interventions to improve preterm birth and highlights the need for an intersectoral approach that addresses the multifaceted challenges of prevention and care for preterm birth, focusing on equity and rights, education, economy, environment (including nutrition and climate) and emergencies (“the five Es”) [21]. 

The seventh and final paper outlines a forward-looking call to action, highlighting investment, implementation in partnership with women and families, integration, and innovation to drive progress [19]. 

Three additional commentaries are also included in the supplement: the first is a high-level political commentary by prominent signatories, including three heads of agencies and the PMNCH Board chair [22], the second focuses on community voices; [13] and the third highlights data and findings on preterm births from recent work led by Countdown to 2030 and partners, placing preterm birth within the context of the maternal-newborn-stillbirth transition framework and the *Every Woman Every Newborn Everywhere* coverage targets [23].

## Progress is possible

The Born Too Soon movement, the report and these papers, take stock of the journey of the past decade – the good and the bad, the challenges and the opportunities to accelerate preterm birth prevention and improved neonatal care into the next decade. Born Too Soon shines a spotlight on countries’ achievements and innovations that can inspire and inform faster progress. The agenda for preterm birth is central to the SDGs, recognising that progress on maternal and newborn health and stillbirths depends on collaboration across sectors. Highlighting where progress is happening, this supplement looks to the future to reduce the burden of preterm birth by investing more in high-quality, respectful care for women and babies so that they can survive and thrive, in every country, in the decade to come.

## Data Availability

All data is available in the paper or in supplementary files. Additional information is available at www.borntoosoonaction.org.
